# The impact of DocosaHexaenoic Acid supplementation during pregnancy and lactation on Neurodevelopment of the offspring in India (DHANI): trial protocol

**DOI:** 10.1186/s12887-018-1225-5

**Published:** 2018-08-04

**Authors:** Shweta Khandelwal, M. K. Swamy, Kamal Patil, Dimple Kondal, Monica Chaudhry, Ruby Gupta, Gauri Divan, Mahesh Kamate, Lakshmy Ramakrishnan, Mrutyunjaya B. Bellad, Anita Gan, Bhalchandra S. Kodkany, Reynaldo Martorell, K. Srinath Reddy, Dorairaj Prabhakaran, Usha Ramakrishnan, Nikhil Tandon, Aryeh D. Stein

**Affiliations:** 1 Public Health Foundation of India, 47, Sector 44, Gurugram, Haryana India; 2 Centre for Chronic Disease Control, Gurugram, India; 3 KLEU’s JN Medical College, Belgavi, India; 4 Sangath, Delhi, Goa India; 5 Child Development Centre, Prabhakar Kore Hospital, Belgavi, India; 6 All India Institute of Medical Sciences, New Delhi, India; 7 Research Foundation, KLE University, Belgavi, India; 8 Hubert Department of Global Health, Rollins School of Public Health Emory University, Atlanta, USA

**Keywords:** Docosahexaenoic acid (DHA), Omega 3 fatty acids, Polyunsaturated fatty acids, n3PUFA, Neurodevelopment, Development assessment scale for Indian infants (DASII), Maternal supplementation, Pregnancy outcomes, Newborn outcomes

## Abstract

**Background:**

Evidence suggests a strong association between nutrition during the first 1000 days (conception to 2 years of life) and cognitive development. Maternal docosahexaenoic acid (DHA) supplementation has been suggested to be linked with cognitive development of their offspring. DHA is a structural component of human brain and retina, and can be derived from marine algae, fatty fish and marine oils. Since Indian diets are largely devoid of such products, plasma DHA levels are low. We are testing the effect of pre- and post-natal DHA maternal supplementation in India on infant motor and mental development, anthropometry and morbidity patterns.

**Methods:**

DHANI is a double-blinded, parallel group, randomized, placebo controlled trial supplementing 957 pregnant women aged 18–35 years from ≤20 weeks gestation through 6 months postpartum with 400 mg/d algal-derived DHA or placebo. Data on the participant’s socio-demographic profile, anthropometric measurements and dietary intake are being recorded at baseline. The mother-infant dyads are followed through age 12 months. The primary outcome variable is infant motor and mental development quotient at 12 months of age evaluated by Development Assessment Scale in Indian Infants (DASII). Secondary outcomes are gestational age, APGAR scores, and infant anthropometry. Biochemical indices (blood and breast-milk) from mother-child dyads are being collected to estimate changes in DHA levels in response to supplementation.

All analyses will follow the intent-to-treat principle. Two-sample t test will be used to test unadjusted difference in mean DASII score between placebo and DHA group. Adjusted analyses will be performed using multiple linear regression.

**Discussion:**

Implications for maternal and child health and nutrition in India: DHANI is the first large pre- and post-natal maternal dietary supplementation trial in India. If the trial finds substantial benefit, it can serve as a learning to scale up the DHA intervention in the country.

**Trial registration:**

The trial is retrospectively registered at clinicaltrials.gov (NCT01580345, NCT03072277) and ctri.nic.in (CTRI/2013/04/003540, CTRI/2017/08/009296).

## Background

The period from conception through to a child’s second birthday, commonly referred to as ‘the first 1000 days’, is a crucial window to improve maternal child health and nutrition indicators and optimize human capital [1, 2]. The brain develops rapidly through neurogenesis, axonal and dendritic growth, synaptogenesis, cell death, synaptic pruning, myelination, and gliogenesis [3–5]. These ontogenetic events build on each other, such that even small perturbations can have long-term effects on the brain’s structural and functional capacity [6]. A mother’s nutrition during this critical phase impacts both prenatal and postnatal growth and the development of offspring [7, 8]. Higher omega 3 long chain poly unsaturated fatty acid (n-3 LCPUFA) levels such as docosahexaenoic acid (DHA) have been associated with enhanced infant neurodevelopment [9–16].

DHA is an important structural component of human brain and retina. It can be obtained from marine algae, fatty fish and marine oils. Since Indian diets are largely deficient in DHA-rich sources, the population levels of plasma DHA are quite low [17, 18]. Indians do consume sources of the precursor, alpha-linolenic acid (ALNA 18:3n-3; a short-chain omega-3 fatty acid) like mustard oil, soybean oil, flaxseeds, walnuts. However, excess omega-6 fats in Indian diets inhibit the endogenous synthesis of DHA from ALNA [19]. Thus, negligible DHA-rich products coupled with an excess of omega-6 sources result in low plasma DHA and a sub-optimal omega-3 to omega-6 ratio among Indian populations [20, 21]. The ideal ratio of omega-3 to omega-6 should be 1:1 to 1:2.5. However, in Indian diets it is usually around 1:17 to 1:20 [22, 23]. These low levels become a concern especially during peak spurts of neurodevelopment such as the first 1000 days [24].

### DHA and neurodevelopment

Long chain polyunsaturated fatty acids, particularly DHA (22:6n-3) and arachidonic acid (20:4n-6), are integral to fetal, neural and retinal development and accrete extensively in the last trimester of pregnancy [25]. Despite the implications for child development, there have been few studies which have comprehensively tested interventions in humans. Makrides et al. showed that high-DHA (approximately 1% total fatty acids) enteral feeds compared with standard DHA (approximately 0.3% total fatty acids) from day 2 to 4 of postnatal life showed significantly higher (unadjusted mean difference, 4.7; 95% CI, 0.5–8.8; adjusted mean difference, 4.5; 95% CI, 0.5–8.5) Bayley’s Mental Development Index scores in Australian girls, however among the boys, it did not differ between groups [26, 27]. Ramakrishnan et al. [28] summarized studies in this area and reported that observational studies indicated a direct association between poor n–3 fatty acid status and increased risk of maternal depression and childhood behavioral disorders such as attention-deficit hyperactivity disorder (ADHD). It has also been hypothesized that prenatal exposure to DHA may also affect later development through fetal programming of the central nervous system and various other physiologic pathways. Ramakrishnan et al. in 2016 reported a beneficial impact of prenatal DHA supplementation on attention in preschoolers at 5 years of age where DHA group children recorded significantly fewer omissions (< 40) as compared to the control group on K-CPT (Conners’ Kiddie Continuous Performance Test). Also, the magnitude of positive association between home stimulation and cognitive functioning was found to be less in the DHA group (b = 0.71; 95% CI: 0.13, 1.29; placebo group: b = 1.71; 95% CI: 1.09), [29]. Another randomized trial, the DHA Intake and Measurement of Neural Development (DIAMOND) study [16] suggested some benefit of DHA supplementation of infants form 1–9 days of age till 12 months on visual acuity of infants at 1 year of age. It was observed that infants fed with control formula had significantly poorer visual acuity than the infants who were fed with DHA-supplemented formulas (*P* < 0.001). Evidence for association between increased consumption of sea food in pregnancy and improved neurodevelopmental outcomes in their children have also been shown in observational studies [30–33]. However, evidence from intervention trials from low- and middle-income settings is scanty. Most of the studies reviewed were conducted in developed countries. Some trials had small sample sizes too. Thus large, well-designed, community-based prevention trials from developing countries are warranted.

In summary, studies conducted to date suggest that improvements in DHA levels in mother may confer some benefit for child neurodevelopment. Furthermore, DHA appears to be safe, with no adverse birth outcomes related to DHA supplementation observed in low- risk pregnancy cases [34]. Very few studies to date have continued supplementation through lactation. We therefore implemented a large scale randomized trial to study the effects of pre- and post-natal DHA supplementation on birth weight, gestational age and neurodevelopment in India, a country with low DHA intakes and a high dietary n-6 to n-3 ratio. This will be the first to examine the effects of in-utero and early life DHA exposure (through maternal supplementation from mid-pregnancy through 6 months postpartum) on postnatal neurodevelopment and body-size of Indian infants. Long term contact and follow-up with this cohort is being planned. The biological specimens being collected (blood, cord-blood, and breast-milk) from the mother-child dyads can further help pursue new hypotheses and unravel critical information about early DHA intervention on later life of an individual [35–41].

## Methods

### Study design

DocosaHexaenoic Acid supplementation during pregnancy and lactation on Neurodevelopment of the offspring in India (DHANI) is a double-blinded, randomized, placebo controlled trial being conducted in India. Study participants are healthy pregnant women and their offsprings.

DHANI will assess the impact of 400 mg of pre- and post-natal DHA supplementation (from ≤20 weeks of gestation to 6 months postpartum) on infant neurodevelopment at 6 and 12 months as measured by the Development Assessment Scale for Indian Infants (DASII). Secondary objectives of this trial include an assessment of the impact of maternal DHA supplementation on: gestational age, new born anthropometry (birth weight, length and head circumference) and APGAR score; infant anthropometry (birth weight, length and head circumference) at birth, 1 month, 6 months and 12 months; number of unfavorable pregnancy outcomes (still births, low birth weight babies, preterm babies); morbidity patterns through 12 months in the two groups. The first mother was enrolled on 6 Jan 2016 and the last on 31st Aug 2017. The data being collected under the trial for mother-infant dyads at each time point has been presented in Table 1.

**Table 1:** Data being collected under DHANI trial

Domain	Measure	Data obtained on woman						Data obtained on child		
		Pregnancy		Delivery	Post partum/ lactation			Birth	6 months	12 months
		Enrolment at < =20 weeks	Each month until Delivery	Delivery	2-3rd Day Post partum	1 month Post partum	6 month post partum			
Socio-economic and demographic profile	Demographics	X								
	family details	X								
	Income	X								
	Education	X								
	Occupation	X								
Medical and Obstetric History	Clinical Investigation	X	X	X	X		X	X	X	X
	Obstetric History	X								
	Complications	X	X	X	X	X	X	X	X	X
	Adverse events	X	X	X	X	X	X	X	X	X
	Eligibility	X								
Blood	Fatty acid composition	X		X			X	X	X	X
Breast milk (30%)	Fatty acid composition				X	X	X			
Body Size & composition	Anthropometry (weight, length, mid upper arm circumference, head circumference of offspring)	X	X	X	X	X	X	X	X	X
Behavioral Factors	Dietary Intake	X							X	X
	Physical activity	X					X			
	Prenatal Care	X	X							
	Substance use	X								
Child development	APGAR							X		
	Developmental Quotient								X	X
	Morbidity	X	X	X	X	X	X	X	X	X

### Study population

The study population consists of 18–35 year old women with singleton pregnancy of ≤20 weeks of gestational age registered at the Obstetrics and Gynaecology outpatient department (OBGYN OPD), Prabhakar Kore hospital (PKH) for antenatal check-up. The PKH under KLE University’s Jawaharlal Nehru Medical College (JNMC) in Belgavi, Karnataka, India caters to the local population and the villages in and around Belgavi. PKH reports nearly 500–600 deliveries per month. Detailed inclusion and exclusion criteria are provided in Table 2.

**Table 2:** Inclusion and Exclusion Criteria for DHANI trial

Inclusion Criteria:	Exclusion Criteria:
• Pregnant woman aged 18 years to 35 years (singleton) at ≤20 weeks of gestation (calculated from the last menstrual period or by ultrasound in 1st trimester as suggested by study physician/team). • Willing to participate in the study and provide all measurement for self, husband and the offspring including anthropometry, dietary assessment and questionnaire plus the biological samples (blood, breast milk) • Willing to provide signed and dated written informed consent. • Plans to deliver at the study hospital • Willing to comply with study specific procedure/instruction	• Women with high-risk pregnancies or bad obstetric history • Women with chronic conditions like Hypertension, heart disease, Cancer, Diabetes, epilepsy, liver disorders, thyroid problem, known history of bleeding disorders or thrombosis or any other medical condition which may affect the safety of mother/infant in opinion of study investigator/physician. • Women allergic (if aware) to any of the test products • Women consuming omega-3/DHA supplements or having used these in 3 months preceding the intervention period. • Participated in another drug trial within or before 3 months from the date of screening under this study or during the study

**Table 3:** Expected Adverse Events (AE) and Serious Adverse Events (SAE)

Averse Events	Serious Adverse Events
• Preterm labour • Premature rupture of membranes • Preeclampsia • Urinary Infection	• Abortion • Abruptio-placenta • Intra Uterine Death • Fresh Still Birth • Macerated Still Birth • Congenital anomalies • Neonatal death • Maternal Death

### Exposure

The exposure is 400 mg of algal DHA to be consumed daily by pregnant women from ≤20 weeks of gestation until 6 months postpartum recruited under the trial. The study staff delivers a bottle with 35 capsules (2 capsules per day X 15 day + 5 extra for spillage, spoilage, etc.,) which match the allotted code of the subject. The bottles are either collected by the participant every fortnight or distributed by study personnel during fortnightly visits at the participant’s home or workplace. The women are instructed to take two capsules daily, preferably at the same time each day.

### Control

The control is a placebo which contains 200 mg per capsule of corn/soy oil (both active and placebo capsules provide the same, but negligible, energy). The placebo capsules provide no DHA and a negligible amount of omega-6 fatty acids. The active and placebo capsules are identical with respect to taste and appearance and only differ in coding.

### Outcomes

#### Primary outcome

- Mean difference in the infant neurodevelopmental score (DQ), motor score and mental scores, as measured by the DASII scale 12 months.

#### Secondary outcome(s)

- Difference in proportions of infants with developmental delay between the DHA and placebo group at 12 months. Developmental delay will be defined as a DASII score ≤ 70.
- Mean difference in infant size (weight, length and head circumference) and APGAR score at birth.
- Mean change in infant size (weight, length and head circumference) at birth, 1 month, 6 months and 12 months between the DHA and placebo group.
- Mean difference in number of still births, preterm and low birth weight babies between DHA and placebo groups.
- Difference in infant morbidity patterns (types of illness, frequency and duration of specific conditions) between DHA and placebo group.

#### Tertiary outcomes

- Difference in DHA values (as measured by fatty acids in maternal blood) from baseline to delivery and 6 months post-partum in the two treatment groups.
- Difference in DHA values (as measured by fatty acids in cord blood and breast milk) in the two treatment groups.

### Ethical approvals

Ethical clearances were obtained from all participating institutions including: Chronic Disease Control (CCDC-IEC_04_2015), Public Health Foundation of India (TRC-IEC-261/15), Jawaharlal Nehru Medical College (MDC/IECHSR/2016–17/A-85) and All India Institute of Medical Sciences (IEC-28/17.11.2015). The trial is registered retrospectively at the clinicaltrials.gov (NCT01580345, NCT03072277) and ctri.nic.in (CTRI/2013/04/003540, CTRI/2017/08/009296). Trial registration was completed after participant recruited had started. An independent Data and Safety Monitoring Board (DSMB) was constituted before the start of the trial to review the study data periodically to monitor safety and outcomes.

### Effect size, power and sample size

The primary outcome variables are infant motor and mental development quotient at 12 months of age. The smallest effect sizes of interest were 0.25 S.D. for motor and mental development at 12 months of age [42]. Using a two-tailed test and a significance level of α = 0.05, a final sample of 338 infants per group (676 mother-child dyads) was required at the end of the study (12 months postpartum) to provide 90% power. A total of 957 mothers have been recruited in the present study, allowing for potential attrition due to serious adverse events, withdrawal, and migration out of the study area. Breast-milk samples are being collected within 3–4 days after delivery and at 1 month and 6 months postpartum from a 30% convenience sub-sample - 150 mother-infant pairs per group, which will have 95% power to detect effect sizes of 0.5 S.D. in DHA levels.

### Recruitment

The pregnant women were screened by the project officer with the help of the attending medical doctor to verify eligibility. A project officer then explained the study in detail and invited the eligible candidate for participation. Written informed consent was provided by all willing participants in presence of a family member. The copy of the informed consent and subject information sheet were provided in their preferred local language (Kannada, Marathi or Hindi).

### Randomization procedure

Consenting women were randomized to their treatment group by the project officer. The randomization scheme consisted of a sequence of blocks such that each block contained a pre-specified number of treatment assignments in random order. The block size of 2, 4 and 6 was used for treatment allocation. The randomization code list was pre-generated for 1200 women randomly allocating 600 participants to the DHA and 600 participants to placebo group. The assignment codes were placed in sealed envelopes at the beginning of the study, and these envelopes were kept sealed at the host institution by a staff-member not involved in the trial.

### Data collection

- Primary outcome variable: The development quotient (DQ) among infants at 12 months of age assessed using the standardized DASII scale [43, 44] by two trained psychologists is the primary outcome variable [45, 46]. DASII is the Indian modification (done in 1970 and 1977) of the Bayley Scale of Infant Development (BSID) using Indian norms for 67 motor and 163 mental items of the BSID. DASII provides a measure of DQ among Indian infants below 30 months of age [47, 48]. DQ is defined as the ratio of functional to chronological age. Third, 50th and 97th percentile norms are given. The maximum DQ score is 100; ≥85 is normal; 71–84 is mild to moderate delay and developmental delay is defined as DQ ≤70 (≤2SD). Median reliability index for motor and mental scales based on correlation between consecutive months is noted to be 0.88 for motor scales and 0.91 for mental scales. The motor development items cover the child’s development from supine to erect posture, neck-control, locomotion etc. It also includes the record for manipulative behaviour such as reaching, picking-up, handling things etc. The mental development items record the child’s cognizance of objects in the surroundings, perceptual pursuit of moving objects, exploring them to meaningful manipulation. It also covers the development of communication and language comprehension, spatial relationship and manual dexterity, imitative behavior and social interaction etc. Each child undergoes DASII assessment twice – once at 6 months and again at 12 months. For infants born preterm, the assessment is done at their corrected ages of 6 and 12 months.

- Secondary outcomes:
- Birth outcomes – Data are collected by a research assistant from hospital records within 24 h after delivery. In case of night deliveries, records from hospital staff are obtained the next morning. All hospital staff were apprised of the variables being collected, procedures and data collection methods before the start of the trial. A periodic (every 6 months) refresher training has also been provided to the staff. The data include whether the birth was a live birth, sex of baby, type of delivery, and anthropometric measurements. Birth weight is measured to the nearest 10 g by using a digital pediatric scale. Low birth weight is defined as recorded birth weight less than 2500 g. Birth length and head circumference are measured by trained hospital staff to the nearest 1 mm using a portable anthropometer with a fixed headpiece and a flexible tape, respectively, according to standard procedures. Gestational age at birth in days is determined based on the dating ultrasound. Preterm delivery is defined as delivery after 20 weeks and before 37 weeks of completed gestation. Foetal losses during pregnancy – including miscarriages/abortions and still-births are recorded by study personnel on site or details are brought by field workers (in case mother went to any other hospital). Stillbirths are defined as fetuses delivered at 20 weeks of gestation or later with no signs of life and recorded as occurring before or during the onset of labor; neonatal deaths are defined as deaths among live-born infants occurring within 28 days after delivery.
- Infant weight, length and head circumference at 6 and 12 months are measured during their scheduled PKH visits. Questions about infant’s health are asked by the field-staff during home-visits (postpartum). Copies of the health-reports from the child’s paediatrician (whether at PKH or elsewhere) are collected. After the screening procedure, at the time of recruitment, data for contact information, maternal obstetric medical history, demographic details and socio-economic status also have been collected by the trained research assistant using pre-tested questionnaires. Anthropometric measurements of both mother and father have been recorded. Dietary intakes of fatty acids were evaluated using a previously validated food-frequency questionnaire adapted for use in pregnant women (recall period- past 3 months) also have been recorded.
- Fatty acid profile: Fatty acid composition of RBC membrane phospholipids are appropriate biomarkers of fatty acid status and reflects dietary intakes. Linoleic acid (18:2n-6); Alpha-linolenic acid (18:3n-3); Arachidonic acid (20:4n-6); Eicosapentanoic Acid (20:5n-3); DHA (22:6n-3) will be measured for this study. Lipids will be extracted form the RBC membrane, phospholipid fraction will be separated by thin layer chromatography (TLC), esterified by procedure described by Lepage and Roy [49] and subjected to gas chromatography (GC) for separation and identification. Non fasting maternal blood samples (5 ml) were obtained by veni-puncture at recruitment, delivery and 6 months postpartum. Neonatal blood samples were obtained from the umbilical cord vein immediately after delivery using the syringe method [50]. A 2 ml venous blood sample was obtained 12 months of age from infants. All samples were collected into tubes containing disodium ethylene diamine tetraacetic acid (EDTA). Plasma and RBCs were separated by cold centrifugation at 800 g for 10 min. Plasma was aliquoted and stored at − 80 °C for later analysis.
- Breast-milk polyunsaturated fatty acids (PUFAs): Breast-milk samples (1 day, 1 month and 6 months postpartum) are taken from 30% random sub-sample. The milk-samples are from a morning feed but not the first one, between 8 and 12 o’clock at PKH. Infants are allowed to suckle the nipple for a few minutes, and then a breast-milk sample (10 ml) is expressed (manual, by mothers themselves) and the feeding continued [51]. The samples are refrigerated immediately (to prevent bacterial growth) and later aliquoted into smaller containers, filled nearly to the top to minimize oxidation, and frozen at − 80 °C until analysis. Before storage, butylated hydroxytoluene is added to a final concentration of 75 μg/mL to prevent lipid oxidation. Since breast-milk PUFA will be expressed as a percent of total fatty acids, complete breast expressions are not required. The samples will be analysed by gas chromatography using standard methods [52].

### Training of staff and calibration of equipment

The personnel for DHANI trial were recruited from local areas and trained before the start of the trial and refresher training was provided every 6 months thereafter. The training imparted an understanding of the trial objectives, study procedures, data collection methods, supplement distribution; recording in the requisite case record forms (CRFs), etc. The personnel used local language for communication with the participants and their families on site (Kannada, Marathi, Hindi).

Standardization (calibration) of measuring instruments is done every 4 months by checking the measuring instruments against an accurate standard to determine any deviation and to correct errors.

### Database and data management

The study database was designed in Microsoft Access based desktop application. The database developed is 21 CFR Part 11 compliant, i.e. it has an inbuilt audit trail function to capture each and every data entry activity, records details of users who access the database with date and time and electronic signature. The scanned forms are received via secured FTP server at the study central coordinating centre in New Delhi, where the data are collated, cleaned, and analysed.

### Study intervention

The test supplements were capsules with either algal DHA or placebo (soy/corn oil in 50:50 ratio). Each DHA capsule contains 200 mg of DHA derived from an algal source. The placebo capsules contained an equal amount of corn/soy oil. All participating women received either 2 placebo or DHA capsules from ≤20 weeks of gestation until 6 months postpartum. The total dosage of DHA being given via active capsules was 400 mg/d (2 X 200 mg). The active and placebo capsules used were identical with respect to taste and appearance and only differed in coding.

#### Manufacture

Identical capsules for the intervention and control groups were provided by DSM Nutritional Products, Mumbai. They were received by the bottling vendor who packaged the batch into white opaque bottles with 15 days supply (30 plus 5 extra for spillage = 35) in each. These bottles were coded the bottling warehouse by staff not involved directly in the study. The supplements were then couriered to the site and provided to women by trained fieldworkers during fortnightly visits at the participant’s home or workplace. The shelf life of each batch of capsules is close to 2 years at room temperature (25 °C).

#### Administration

A fortnightly schedule was drawn up for each woman from the day of her randomization. Supplements were provided by field workers during home visits. If informed about travel plans by the participant, study staff provided more than 1 bottle at a time. The women were instructed to take two capsules daily, preferably, at the same time each day. All the women also received iron-folic acid tablets for 100 days as per the Government of India policy.

### Monitoring adherence

Compliance for each woman was recorded in a basic form (15 days record) in which the participant marked with a tick if she consumed the capsule. In case she expressed any difficulties completing the form, the FW followed up by asking her details during home visits. The 2 weeks’ record along with the left over/unused capsules in the bottle of the supplements was collected by field workers from the participants’ home. During the home visit, the field worker also recorded details of any side effects, adverse event or illness and capsule count in a follow-up record form (Compliance Form). Weekly reminder phone calls were done for each mother by study personnel. Compliance is calculated as the total number of capsules actually consumed, expressed as a percentage of the total number expected to be consumed.

### Blinding

All study participants and members of the study team were blinded to the treatment scheme throughout the intervention period of the study. Data will be unblinded for the analytical study team after the last baby born in the study turns 1 year of age. Since the study may be extended for follow-up of child development, participants and field personnel will remain blinded to the treatment allocation.

### Emergency unblinding

Unblinding will only be performed if the number of serious adverse events (SAEs) observed during the trial is significantly higher in one group than the other. The final discretion to make this call will that be of the DSMB.

### Reporting of adverse events

Adverse events (AEs) or SAEs (hospital admission, maternal or fetal or child death, abruption placenta, congenital anomaly, any other medically significant event) are reported as soon as (within 24 h of knowing) possible by the project officer to the study physician, who in turn examines and notifies the site principal investigator about the nature and severity of the AE. The study’s site principal Investigator (PI) notifies all serious AEs within 24 h to the trial Principal Investigator, Institutional Review Boards (IRBs). The DSMB is also notified each time. A summary quarterly report to DSMB and annual report to the ethics committees is also being submitted for their perusal. Table 3 lists the expected AEs and SAEs.

### Compensation

Clinical trial insurance has been procured to compensate clinical trial participants where required (Policy Number OG-17-1401-3306-00000001). Also, the participants are being compensated for their travel expenses when they are called for follow up assessments.

#### CONSORT statement

**Fig. 1:**
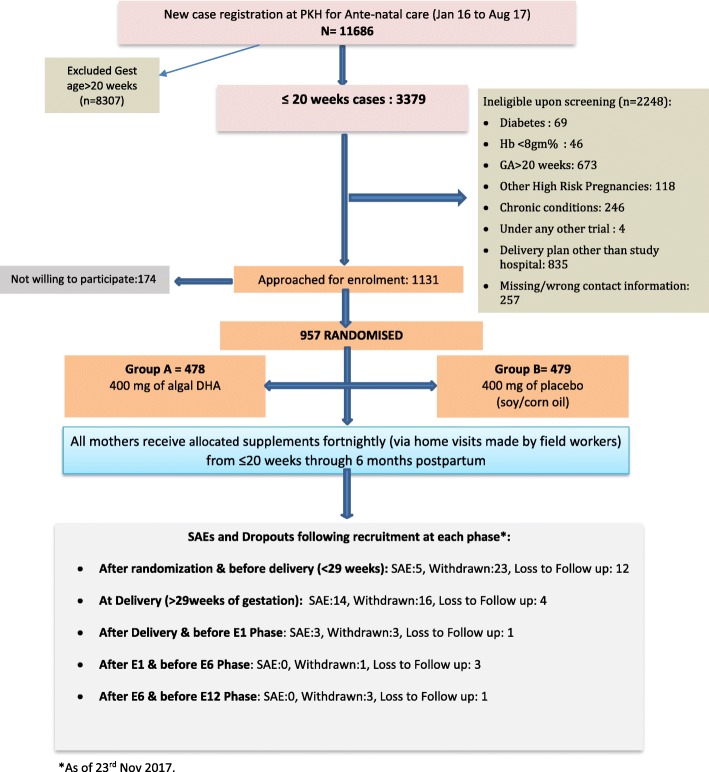
CONSORT Diagram

All pregnant women screened for eligibility in this trial will be accounted for and a CONSORT statement will be prepared (http://www.consort-statement.org). Reasons for early withdrawal will be listed for all participants who prematurely discontinued treatment or left the study. The number of participants who were screened eligible but not randomized are presented and the reasons for non-participation (where available) are recorded. A diagram mapping the flow of participants to date is provided (Fig. 1- CONSORT diagram). Recruitment started on 6th Jan 2016 and ended on 31st Aug 2017. We had expected to enroll 1000 women by March 2017 but were delayed by an unexpected capsule shortage, as a result of which we could not recruit for about 4 months. The final number recruited and randomized is 957. About 70% of the enrolled mothers have delivered so far and 15% have completed the trial.

### Statistical analyses

All the analyses will be performed on the principle of ‘intention to treat’ unless otherwise specified (i.e., we will compare patients in the groups to which they were originally randomly assigned). Statistical analyses will be performed using STATA Version 14.0. We will summarize maternal and household characteristics in the two groups and assess the effectiveness of randomization by comparing these between the groups. Continuous, normally distributed variables will be summarized as means and standard deviations, while skewed variables will be summarized as medians and inter-quartile ranges. Categorical/binary variables will be summarized using proportions. Comparisons on baseline characteristics will also be made between the final study sample and those who were lost to follow up.

For the primary outcome, we will test the unadjusted difference in the mean DASII score at 6 months and 12 months between the two treatment groups using a two-sample t-test. For adjusted analyses, we will consider the DASII score as a continuous variable and use a multiple linear regression model, adjusting for treatment, sex of the child, maternal age, maternal education, socio economic status, birth-weight and other factors which we find out to be significant at baseline comparison. We will adjust for the 6 month DASII score in the model examining the difference in score at 12 months between the treatment groups using Generalized Estimating Equation (GEE) analyses to account for correlation between the 6 month and 12 month DASII score.

The DASII score will also be analysed as a binary variable. DASII score ≤ 70 will be used to defined developmental delay. The unadjusted and adjusted relative risk in the proportion of DASII score (≤70) at 12 months between DHA and placebo groups will be calculated using a log-binomial regression. In case of convergence issues with the log-binomial model, logistic regressions will be used to conduct the adjusted analyses and adjusted odds ratios will be reported instead of adjusted relative risks.

The differences in weight, length, head circumference, still birth, Apgar score at 1 min & 5 min of the infants at birth will be tested using t-test. Adjusted analyses will be performed using multiple linear regression.

For longitudinal analysis of weight, length and head circumference (i.e. at birth, 1 month, 6 month and 12 month), we will also compare the average growth trajectories of the infants between the treatment and placebo groups using Generalized Estimating Equation (GEE) models. The adjusted analyses will also be performed adjusting for time, maternal weight and height, breast-feeding practices and maternal dietary intake.

### Subgroup analyses

The following apriori subgroup analyses will be carried out to evaluate potential heterogeneity of effect:

- Maternal Age (18–20; 21–25;26–30;31–35 years)
- BMI categories(< 23;≥ 23 Kg/m^2^)
- Parity
- Gravidae (primi versus multi)
- Dietary patterns (Vegetarian versus non-vegetarian diet)
- Physical activity (Moderate versus sedentary)
- Gestational age (< 32; 30–32; 32–35;35–38; ≥38 weeks)
- Compliance (< 80%, 80–90%,> 90%)
- Duration of supplementation

The results of these subgroup analyses will be treated with caution as this study is not powered for this analyses. In each subgroup analyses, a model will include the subgroup variable along with its interaction with treatment. A test of whether the treatment affects across the levels of the subgroup will be constructed by assessing the significance of the interaction.

### Missing data

**S**ensitivity analyses will be performed using complete case analysis and multiple imputation for missing data to evaluate the potential effect of missing outcomes.

## Discussion

### Implications for public health and nutrition in India

In India scientific evidence from iron-folic acid supplementation during pregnancy has been translated to supplementation policy and substantially influenced government actions. More novel dietary interventions which could benefit offspring birth outcomes and enhance later life growth and development need to be explored. This protocol describes a randomised controlled trial comparing the effect of DHA (400 mg/day) with placebo on the neurodevelopment of infants born to pre- and post-natally supplemented mothers. Both animal and human observational studies and few clinical trials point to the possibility that DHA may have a role in the enhancement of offspring neurodevelopment. There have been no randomised controlled trials designed specifically to determine the effect of DHA on neurodevelopment in India. To the best of our knowledge this is the first such randomised controlled trial conducted anywhere in the world which starts supplementation during pregnancy and continues through 6 months post-partum. This trial will lead to enhanced understanding of the role of maternal DHA supplementation on in-utero and early-life cognitive and motor development among their infants. Results from this study will provide the first high quality evidence on whether a prenatal and continued as postnatal DHA supplement improves the neurodevelopment of 1 year old infants born to supplemented mothers.

Although the mechanisms involved are not completely understood, the active properties of DHA are thought to include effects on neuronal development and plasticity, receptor-mediated signaling, changes in membrane fluidity, the formation of second messengers, and/or enhancement of the production of anti-inflammatory lipid mediators due to the availability of DHA as substrate [34, 53].

If successful, we will work to ascertain the best ways to translate the findings to the existing infrastructure and delivery mechanisms of national child development and nutrition programs like the Integrated Child Development Scheme, Anganwadi workers, ASHAs etc.

## Abbreviations

APGAR: Appearance, Pulse, Grimace, Activity, Respiration
CCDC: Centre for Chronic Disease Control
DHA: Docosahexaenoic Acid
DSMB: Data and Safety Monitoring Board
FW: Field worker
JNMC: Jawaharlal Nehru Medical College
PKH: Prabhkar Kore Hospital
PO: Project Officer
PUFA: Poly unsaturated fatty acids
